# The impact of facilitation in a problem-based pedagogy on self-directed learning readiness among nursing students: a quasi-experimental study in Tanzania

**DOI:** 10.1186/s12912-021-00769-y

**Published:** 2021-12-06

**Authors:** Walter C. Millanzi, Patricia Z. Herman, Mahamudu R. Hussein

**Affiliations:** 1 Department of Nursing Management and Education, School of Nursing and Public Health, The University of Dodoma (UDOM), Dodoma, United Republic of Tanzania; 2 Department of Administration and Hospital Management, Rabininsia Memorial Hospital, Dar es Salaam, United Republic of Tanzania; 3 Department of Pediatric Nursing, Alexandria University, Alexandria, Egypt

**Keywords:** Learning readiness, Problem-based pedagogy, Nursing students, Self-directed learning

## Abstract

**Background:**

Self-directed learning is important in nursing as it is associated with improved clinical and moral competencies in providing quality and cost-effective care among people. However, unethical professional conduct demonstrated by some graduate nurses is linked with the way they are developed in schools alongside the content and pedagogies prescribed in nursing curricula. Pedagogical transformations appear to be inevitable to develop enthusiastic nursing students who can work independently in delivering quality and cost-effective nursing services to people. This study intended to examine the impact of facilitation in a problem-based pedagogy on self-directed learning readiness among undergraduate nursing students in Tanzania.

**Methods:**

A controlled quasi-experimental design was conducted in Tanzanian higher training institutions from January to April 2019. A 40-item Self-directed learning Readiness scale for nursing education adopted from previous studies measured self-directed learning and the Student A descriptive analysis via a Statistical Package for Social Sciences software program (version 23) was performed to establish nursing students’ socio-demographic characteristics profiles. Independent samples t-test determined mean scores difference of self-directed learning readiness among nursing students between groups while regression analysis was performed to discriminate the effect of an intervention controlled with other co-related factors.

**Results:**

The post-test results of self-directed learning readiness showed that nursing students scored significantly higher [(M = 33.01 ± 13.17; t (399) = 2.335; 95%CI: 0.486,5.668)] in the intervention group than their counterparts in the control. Findings of SDL readiness subscales were significantly higher among students in the intervention including self-management [(M = 10.11 ± 4.09; t (399) = 1.354; 95%CI: 0.173,4.026)], interest learning [(M = 9.21 ± 2.39; t (399) = 1.189; 95%CI: 0.166,4.323)] and self-control [(M = 13.63 ± 5.05; t (399) = 2.335; 95%CI: 0.486,5.668)]. The probability of nursing students to demonstrate self-directed learning readiness was 1.291 more times higher when exposed to the intervention (AOR = 1.291, *p* < 0.05, 95%CI: 0.767, 2.173) than in the control.

**Conclusion:**

Facilitation in a problem-based pedagogy promises to change the spectrum of nursing learning habits potentially to their academic and professional achievements. Nurse tutors need to be empowered with it to prepare nursing students to meet their academic and professional potentials.

## Introduction

As other African countries, Tanzania faces the challenge of inadequate nursing tutors and co-related resources to accommodate the increased enrollment rate of nursing students in nursing schools [1, 2]. Increased enrollment rate further strains academic faculty in nursing schools and clinical instructors in training hospitals’ to manage large class sizes and or groups in facilitating effective learning among nursing students using conventional pedagogies such as lectures, bed tutorials, discussions, assignments, and or demonstrations because they are cheap, easy to implement, can cover an extensive course content at once [3].

Amid huge class sizes alongside the shortage of nursing tutors and or trained clinical instructors, the pedagogical inadequacies in nursing education are partly linked with low self-directed learning among nursing students in both classroom and clinical environments [4, 5]. Self-directed learning is defined here as learning habits demonstrated by an individual in taking charge of his learning activities with minimal assistance from others [6]. A self-directed individual is expected to demonstrate abilities in diagnosing learning needs, formulating learning goals, and identifying resources for learning, choosing and implementing appropriate learning strategies, and evaluate learning outcomes.

Literature unfolds that nursing tutors who are present in training institutions and hospitals are argued to demonstrate teaching skills in the way they were taught during their undergraduate and or postgraduate schoolings [7, 8]. They demonstrate tendencies of focusing to cover huge course content within a short period as scheduled in the school calendar or Almanac [9]. Nursing students developed under conventional pedagogies are believed to be teacher-dependents in their learning activities and thus, they become unable to demonstrate self-directed learning [10]. The situation seems to demand a pedagogical transformation as a backbone in developing nursing students with self-directed learning skills [9].

A pedagogical transformation that develops and implements problem-based pedagogy is reported to provide robust evidence in enhancing group and self-directed learning among learners [11]. Problem-based pedagogy is an educational strategy rooted in a social constructivist theory of learning that uses real-life or hypothetical ill-structured problems as an initiator and motivator of learning among students [12]. Via small groups (5 to 8 members) students work collaboratively after sometimes of individual learning of the assigned problem and share their solutions with colleagues in the entire class.

Under problem-based pedagogy, students take responsibility for their learning activities by identifying learning objectives, resources, and strategies appropriate to solve the assigned problems with minimal support from a teacher. The teacher’s role in a problem-based pedagogy is just to guide students, clarify difficult areas and facilitate resources during their learning processes. Though very few, available literature has demonstrated the effect of problem-based pedagogy on academic and behavioral outcomes among students. The work of Keziah for example has shown that PBL pedagogy enhances students’ learning habits by motivating them to learn biology more than the lecture-based pedagogy did [13].

On the contrary, Karimi and colleagues explored the effect of PBL pedagogy on improving health-promoting behaviors among girl students of which findings revealed that the total girls’ health literacy was significantly improved with higher mean scores after intervention than girls in the control group did [14]. Additionally, other literature on the effect of PBL on self-directed learning (SDL) skills among Physics learners revealed a significant difference in SDL whereas students in the problem-based pedagogy than those in the control group [15]. Students in the problem-based pedagogy demonstrated abilities to develop their learning initiatives and problem-solving strategies than those exposed in the conventional pedagogies.

However, some literature questions whether learners trained using the problem-based pedagogy are well prepared for skills to practice and solve real-life problems as learners trained using conventional pedagogies. Albanese and Mitchell and Vernon and Blake conducted a meta-analysis to evaluate studies that are integrated into a problem-based pedagogy [16, 17]. They argued that the effect of problem-based pedagogy was similarly comparable to the effects of conventional pedagogies in knowledge tests though learners differed in problem-solving skills.

Moreover, a systematic and Meta-analysis by Newman ever reported the effectiveness of problem-based pedagogy in higher education [18]. The report narrated that the existing overviews do not provide high-quality evidence with which to provide robust answers to questions about the effectiveness of problem-based pedagogy in enhancing motivation to learn among students. In the same veil, Sanson-Fisher and Lynagh revealed that “available evidence though methodologically flawed, offered little support for the superiority of PBL over traditional curricula [19]. Nevertheless, Pagander and Jason examined empirical research supporting the effectiveness or ineffectiveness of problem-based pedagogy in medical education. They found contradictory evidence regarding its effectiveness with the majority of support coming from the educational medicine field [20].

Given little evidence from literature about the effect of problem-based learning in enhancing academic and behavioral outcomes among students, there seems no clear and conclusive consensus on the effectiveness of problem-based pedagogy over conventional pedagogies. The controversy about the effect of problem-based pedagogy is also observed in Tanzania where there is misguided knowledge and understanding about how can problem-based pedagogy be developed and implemented to enhance SDL among nursing students in Tanzanian higher training institutions [9, 21].

The available locally scholarly works do not show clearly whether nurse tutors in higher training institutions are implementing problem-based pedagogy in facilitating learning among nursing students. The current study aimed at addressing the pedagogical gaps in nursing by examining the impact of facilitation in a problem-based pedagogy on SDL readiness among nursing students in Tanzania. The study was guided by a null hypothesis that stated “there is no significant difference between nursing students exposed in the facilitation in a problem-based environment pedagogy and their counterparts in the control group in their levels of SDL readiness.

## Methods and materials

### Study design and approach

The study employed a controlled quasi-experimental (pre-post) design with quantitative research approach among 401 randomly selected undergraduate nursing students (Intervention: *n* = 134 students and the control: *n* = 267 students) between January and April 2019. The difference of sample between groups is based on the ratio of 1:3. The study believed that nursing students are exposed to different sources of knowledge including media, experts, and or outside school-educated peers. Thus, comparing one student exposed to the intervention with three students in the control group was adequate to discriminate the effect of an intervention over the control.

### Study location

The study was conducted in higher training institutions in two regions including Dar es Salaam and Dodoma, Tanzania. The distance factor was used to select regions purposively owing to the availability of higher training institutions with nursing programs. One higher training institution was randomly selected by a simple random technique using the lottery method to get two institutions (One higher training institution per region). The institutions were randomly allocated to either be the intervention or control group by a statistician independent to this study. The nature of study locations and the procedures involved in selecting them would minimize selection bias, allocation bias, performance bias and information contamination among undergraduate nursing students.

### Inclusion and exclusion criteria

**Fig. 1:**
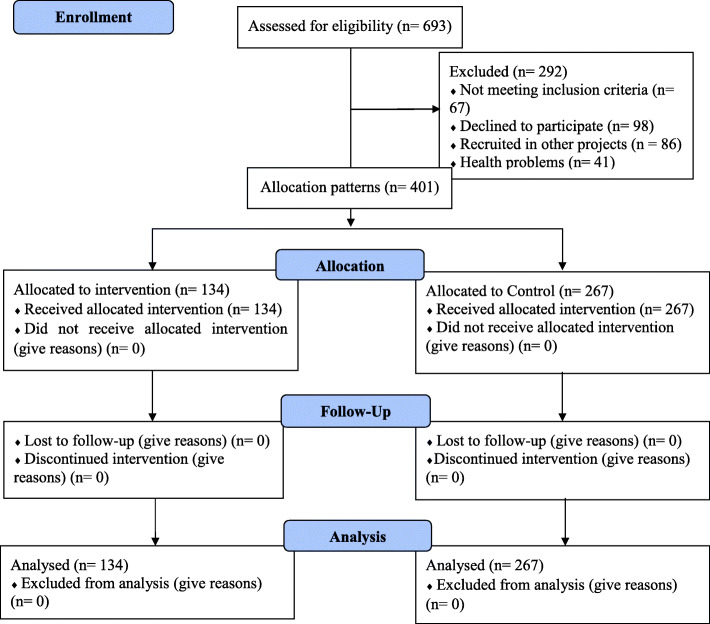
Allocation CONSORT flow diagram representing data collection points for the intervention group and the comparison group

The entrance of students in this study was set voluntarily after they were explained the aim, advantages, and disadvantages of participating in the study. The study included first year undergraduate nursing students enrolled in higher training institutions and who were available and accessible during the study, students who provided informed consent to participate in the study, those who lived in or off-campus, and nursing students who were not recruited in other projects. Students in all training institutions were in the same midterm. On the other hand, the study excluded nursing students with health problems. As shown in Fig. 1, sums of 693 nursing students were eligible to participate in the study. However, 401 (57.8%) consented to join the study whereas 292 (42.2%) were excluded due to different reasons including postgraduate nursing students 67 9.7% (*n* = 67), decline to participate 14.1% (*n* = 98) and recruited in other projects 12.4% (*n* = 86) and having health problems 5.9% (*n* = 41).

Sums of 134 nursing students were assigned to the intervention arm whereas the other 267 nursing students were assigned to the control group. The interventional group learned the conflict resolution lesson materials through facilitation in a problem-based pedagogy (PBP) while the control group learned the the standard conflict resolution lesson materials through a lecture-based pedagogy (LBP) pedagogy. They were then followed for end-line assessment after an intervention. There was no loss to follow-up students in the study.

The study was conducted through four phases including Phase one (for ethical approval and clearance, development of the conflict resolution lesson materials, consultations and meetings with stakeholders, recruiting and training research assistants, and piloting of the research instruments); Phase two (For identifying and sampling study locations, recruiting undergraduate nursing students, and baseline assessment); Phase three (for intervention implementation), and Phase four (for end-line assessment/follow-up). With the technical and professional support from the consulted experts, the principal investigator developed the conflict resolution lesson materials which were then implemented by research assistants’ by using facilitation in a problem-based pedagogy. The interventions were conducted during the early days of a semester as per the study schedule that was blended with the institution’s academic schedule.

### Data collection

Separate unoccupied classrooms in respective higher training institutions were used for the nursing students to fill the questionnaires and for the researcher and research assistant to deliver the conflict resolution lesson materials among nursing students. Undergraduate nursing students were given a brief descriptions of how to fill the questionnaires then the copies were distributed among them. The researcher and assistants were available throughout the process to supervise and address any queries from the nursing students before collecting all copies of the questionnaires and secure them. A schedule of the next meeting to start the intervention was shared with nursing students before moving from the room.

#### Research instruments

The research tool that was used for data collection consisted of two parts including the socio-demographic part and the part that assessed self-directed learning readiness among nursing students. Socio-demographic items included Gender, age, marital status, accommodation, interest, and reasons to join nursing programs, satisfaction in learning nursing programs, perceived benefits, and challenges in learning nursing programs. A study adopted a 40 items 5-points Likert scale Self-directed learning Readiness scale (SDLRS) for nursing education and used by several previous studies to measure self-directed learning among nursing students [22–29].

The tool had scales ranging between 1 = strongly disagree and 5 = strongly agree. It consisted of three domains including students’ self-management (13 items), in which the responses were then transformed statistically into “Yes” response for a weight of “1″ score and “No” for the weight of “0″ with sums of scores ranging from 0 to 13. Mean as a central tendency measurement was used to define the endpoint of analysis of this domain whereas scores above mean were considered good self-management in the learning process. Moreover, responses of the desire for learning (12 items), and self-control domains (15 items) were treated the same as the self-management domain. Sums of scores ranging from 0 to 12 defined the desire to learn while a range of 0 to 15 scores defined self-control during the learning process. Scores above means were considered positive for the desire to learn, and good for self-control during the learning process respectively, otherwise not.

The validity and reliability tests were performed first before subjecting the tool to field data collection. It was shared with the subject matter, and statisticians for appropriateness of the items, consistency, and clarity to fit the literacy level of undergraduate nursing students. The comments were addressed accordingly and a tool was re-shared among the consulted research team for their approval to get the final version. The amended tool was then subjected to a pilot study among 40 nursing students from the training institution other than the sampled ones for testing its reliability. The findings of scale analysis from the pilot study revealed a Cronbach’s Alpha of 0.807, which was acceptable as recommended by previous studies [28, 30, 31].

The first part of the tool assessed students’ socio-demographic characteristics profiles including age, sex, marital status, year of study, accommodation status, interest to join nursing, satisfaction in the nursing program, and a reason to join nursing programs just to mention a few. For analytical purposes, the Likert responses were transformed into dichotomized (“Yes-No”) responses of which “Yes” was associated with an action or behaviors to have been demonstrated by the student and it was assigned a weight of “1″ score otherwise not (“No” response = “0″ score). A new dichotomized variable of self-directed learning was computed whereas a score of > 20 of the total item score was considered to be the acceptable level of SDL readiness, otherwise not.

#### Intervention implementation

The study adopted the problem-based pedagogy framework by Millanzi et al.*,* [9, 21]. The framework includes some stages to implement problem-based pedagogy in facilitating learning among nursing students. They include self-introduction and group formulation, presentation of paper-based scenario, time for resolving the presented problems, debates and sharing of solutions in the class, guidance and support from facilitators, peer and facilitator assessment, and summary of the study activities. Students learned two units including the unit about the concept of conflict resolutions alongside the strategies to address it. The second unit was all about demonstrations of conflict resolutions in nursing.

##### Introduction and group formulation

The first day of the intervention was used by the facilitators to introduce the intervention by providing brief descriptions about the duration of the intervention, learning objectives, and in-out-class learning activities. The groups of 5 to 8 members were randomly formed under the supervision of the researcher and assistants and group leaders were identified including chairpersons and secretaries while other members participated in addressing the assigned activities accordingly.

##### Paper-based scenario presentation, resolving, debates, and sharing (30 min)

The researcher and assistant distributed pieces of papers with a conflicts-based scenario in each group followed by brief descriptions on the ways students were required to address them individually and in their groups. The individual and group learning activities were identified before starting the session and queries from students were addressed by the researcher and assistants accordingly. Learning activities that were assigned to students included the identification of the professional context of the presented scenario, identification of known issues and new ones, issues to know more, and identification of issues to learn from the scenario.

Over a period of 60 to 120 min, nursing students had to clarify, rank, and assign learning tasks among themselves to find appropriate strategies to resolve the presented scenario in their groups. Given the facilitator’s guidance and facilitation, nursing students were supposed to identify and suggest resources necessary in resolving the assigned scenario. After a brief description and assignment of the scenario among groups, students were given one to address the scenario by exploring possible conflict resolution strategies that suit the given problem before being shared and discussed in the entire class during the next scheduled session.

The researcher and assistant were available to receive any calls, queries, and concerns from students either by mobile texts, orally, or in writings through email. This was meant to help them if they would need any help or clarifications about the assigned task. In the next session (after a week) students presented, discussed, and shared what they explored in the entire class. The facilitator’s guided min-debates were allowed whereas groups had to defend their works when other groups challenged and critiqued them. Students were also offered opportunities to share real-world events and other related phenomena related to the topic under study.

##### Support from facilitators and facilitation of groups

Facilitators organized and managed the learning environment by ensuring that there was an adequate supply of learning materials, opening windows to allow fresh air to enter the room, enough light and enough space that facilitated students’ discussions, free movements, and that which minimized disturbances among students. Given the prescribed roles by the researcher and assistant, group leaders were sometimes used to lead, monitor, control, and evaluate group members.

##### Assessment modes

Peer assessment among students was used to determine students’ interactions and abilities to evaluate one another throughout sessions. The student’s experience inventory developed by the researcher was used to evaluate the implementation of conflict resolution material that was developed in the nature of facilitation in a problem-based pedagogy. Using a random selection method, a researcher and assistant selected 2 to 5 students randomly after each session to share their experiences about the sessions. Moreover, the written posttest was used as a summative assessment to measure self-directed learning among nursing students.

### Data analysis

A Statistical Package for Social Sciences (SPSS) software program version 23. Error checking (data screening) test was performed to test for normality before data analysis to identify the type of analysis model to be used. Findings from the normality test indicated data were approximately normally distributed and therefore parametric measurements were opted. All missing data were excluded from the analysis models. Descriptive analysis through chi-square and crosstabulation was performed to establish nursing students’ socio-demographic characteristics profiles and determine the relationship between categorical variables. Independent two-sample t-tests were performed to compare the mean score to establish mean score change and differences among nursing students between groups. Binary and multinomial logistic regression analyses controlled with other co-related factors were performed to determine the association between  an intervention and self-directed learning readiness among nursing students. The confidence interval was set at 95%CI with the power of the study set at 80%. A significance level was set at 5% (0.05) of which < 0.05 of the association between variables was considered statistically significant.

## Results

### Nursing students’ socio-demographic characteristics profiles

For the descriptive purpose, nursing students’ socio-demographic profiles were assessed of their homogeneity between groups. Findings in Table 1 show that students in both groups had similarly comparable characteristics except for the satisfaction in learning nursing program (χ^2^ = 31.660^a^; *p* < 0.01), perceived learning benefits in learning nursing program (χ^2^ = 6.200^a^; *p* < 0.05), and perceived challenges during students’ learning activities (χ^2^ = 9.665^a^; *p* < 0.05). These factors were controlled during analysis to discriminate the effect of an intervention on SDL readiness among nursing students.

**Table 1:** Nursing students’ Socio-demographic characteristics Profiles (*n* = 401)

Variable	Intervention (***N*** = 134)	Control (***N*** = 267)	χ^**2**^(***p***-value)
	n(%)	n(%)	
**Gender**			
Males	83 (61.9%)	181 (67.8%)	0.244^a^(0.721)
Females	51 (38.1%)	86 (32.2%)	
**Age**			
< 24 yrs.	6 (4.5%)	25 (9.4%)	0.192^a^(0.398)
25–29 yrs.	100 (74.6%)	195 (73.0%)	
> 30 yrs.	28 (20.9%)	47 (17.6%)	
**Marital status**			
Single	123 (91.8%)	248 (92.9%)	0.802^a^(0.695)
Married	11 (8.2%)	19 (7.1%)	
**Accommodation**			
In-campus	43 (32.1%)	235 (88.0%)	8.773^a^(0.001)
Off-campus	91 (67.9%)	32 (12.0%)	
**Interest in joining nursing program**			
Yes	92 (68.7%)	204 (76.4%)	2.771^a^(0.096)
No	42 (31.3%)	63 (23.6%)	
**Reasons to join nursing program**			
Own choice	71 (53.0%)	139 (52.1%)	0.430^a^(0.934)
Parent’s/peer pressure	29 (21.6%)	55 (20.6%)	
Easier to get a job	24 (17.9%)	48 (18.0%)	
Entry qualifications	10 (7.5%)	25 (9.4%)	
**Satisfaction in learning nursing program**			
Yes	78 (58.2%)	224 (83.9%)	31.660^a^(0.001)
No	56 (41.8%)	43 (16.1%)	
**Perceived benefits in learning nursing program**			
Yes	104 (77.6%)	233 (87.3%)	6.200^a^(0.013)
No	30 (22.4%)	34 (12.7%)	
**Perceived challenges during learning process**			
Difficult accessing updated learning materials	24 (17.9%)	56 (21.0%)	9.665^a^(0.046)
Complex course contents	49 (36.6%)	74 (27.7%)	
Inadequate support from lecturers	18 (13.4%)	37 (13.9%)	
Limited time	25 (18.7%)	79 (29.6%)	
No conducive environment	18 (13.4%)	21 (7.9%)	

*Source: Field Data (2019)*

Furthermore, Table 1 indicates that sums of 73.6% (*n* = 295) of nursing students were aged between 25 and 29 years while 65.8% (*n* = 265) of them were males. Sums of 69.3% (*n* = 278) of nursing students were accommodated by their training institutions. Nevertheless, the study observed that 73.8% (*n* = 296) and 75.3% (*n* = 302) of nursing students were interested and satisfied with nursing programs respectively. However, some students reported experiencing learning setbacks including difficulties in accessing updated learning materials (20.0%), complex course contents (30.7%), and inadequate support from tutors/lecturers (13.6%), limited time (26.0%), and unconducive learning environment (9.7%).

### Independent samples t-test of the SDL readiness mean score differences among nursing students between groups

As shown in Table 2, there was no significant difference in SDL readiness mean scores among nursing students at baseline (*p* > 0.05). However, post-test results show a significant increase in the overall SDL readiness mean scores set at α = 5% (*p* < 0.05) among nursing students in the intervention group (M = 33.01 ± 13.17) than those in the control group (t = 2.335, p < 0.05; 95%CI: 0.486, 5.668).

**Table 2:** Independent samples t-test of the SDL readiness and its domains mean score differences among nursing students between groups (*n* = 401)

	Pre-test					Post-test				
	Intervention	Control	t-value (399) = −1.354			Intervention	Control	t-value (399) = 2.335		
	M (SD)	M (SD)	***p***-value	95% CI		M (SD)	M (SD)	***p***-value	95% CI	
				Low	Up				Low	Up
**SDL**	22.06 ± 11.90	20.97 ± 11.05	0.082	−0.269	4.453	33.01 ± 13.17	21.94 ± 10.07	0.020	0.486	5.668
**SM**	5.01 ± 2.07	6.00 ± 3.02	**t-value (399) = −1.354**			10.11 ± 4.09	5.71 ± 3.11	**t-value (399) = 1.354**		
			0.301	−0.124	2.354			0.010	0.173	4.026
**LI**	5.83 ± 1.24	5.02 ± 1.01	**t-value (399) = −1.108**			9.21 ± 2.39	6.12 ± 1.50	**t-value (399) = 1.189**		
			0.071	−0.052	2.201			0.031	0.166	4.323
**SC**	7.07 ± 1.12	6.79 ± 1.08	**t-value (399) = −1.027**			13.63 ± 5.05	5.13 ± 1.02	**t-value (399) = 1.631**		
			0.108	−0.105	2.081			0.019	0.646	4.124

*Source: Field Data (2019)*

### Independent samples t-test of the SDL subscales mean score differences among undergraduate nursing students between groups

The post-test findings of the SDL readiness subscales in Table 2 indicate that students’ mean scores of self-management (SM) were significantly higher among those exposed to intervention compared to their counterparts in the control group did (t = 1.354; *p* < 0.05; 95%CI: 0.173,4.026). Moreover, students’ mean scores of learning interest (LI) were significantly higher among those exposed to intervention than their counterparts in the control group t = 1.189; p < 0.05; 95%CI: 0.166,4.323). That is more, students’ mean scores of self-control (SC) were significantly higher among those exposed to intervention against nursing students in the control group (t = 1.631; *p* < 0.05; 95%CI: 0.646, 4.124).

### The effect of the intervention on Self-directed learning, among undergraduate nurse students between groups

Findings of the binary and multiple logistic regression controlled with students’ socio-demographic characteristics profiles in Table 3 indicate that students who were exposed to the intervention were more times likely to demonstrate SDL readiness than their counterparts in the control did (AOR = 1.291, *p* < 0.05, 95%CI: 0.767, 2.173). The finding may imply that the odds of developing SDL readiness among nursing students was 1 times higher among those exposed in the PBP than when they would be exposed to a lecture pedagogy. Moreover, apart from the effect of an intervention, the probability of nursing students demonstrating SDL readiness was more times higher among those who were living in-campus (AOR = 1.688, p < 0.05, 95%CI: 0.995, 2.863). Other co-related factors were not significantly associated with students’ SDL readiness (*p* > 0.05) as shown in the table.

**Table 3:** The effect of an intervention on SDL readiness among nursing students between groups (*n* = 401)

***Variables***	***OR***	***95%CI***		***P-value***	***AOR***	***95% CI***		***P-value***
		***Low***	***Up***			***Low***	***Up***	
***Group***								
Intervention	1.729	1.130	2.646	0.012	1.291	0.767	2.173	0.036
Control (rf)								
Gender								
Male	0.231	0.105	1.339	0.101	0.177	0.024	1.534	0.491
Female (rf)								
Age								
< 24 yrs.	1.121	1.009	1.522	0.112	1.062	0.667	1.859	0.243
25–29 yrs.	0.927	0.131	1.537	0.212	0.419	0.133	0.810	0.298
> 30 yrs. (rf)								
Marital status								
Single	0.619	0.111	0.920	0.100	0.588	0.207	0.873	0.224
Married (rf)								
Accommodation								
In campus	1.958	1.268	3.022	0.002	1.688	0.995	2.863	0.042
Off-campus (rf)								
Interest								
Yes	0.129	0.061	0.306	0.322	0.037	0.009	0.103	0.416
No (rf)								
Reasons to choose nursing program								
Own choice	1.008	0.731	2.202	0.661	0.916	0.367	1.777	0.811
Parent’s/peer pressure	0.551	0.120	1.252	0.812	0.239	0.133	0.537	0.833
Easier to get a job	1.019	0.749	2.005	0.620	1.255	0.844	2.423	0.626
Entry qualifications (rf)								
***Satisfaction in learning nursing***								
Yes	1.572	1.211	2.715	0.111	1.661	1.187	2.014	0.136
No (rf)								
Perceived benefits in learning nursing								
Yes	0.742	0.208	1.434	0.103	0.806	0.357	1.092	0.113
No (rf)								
Perceived challenges during learning								
Accessing updated learning materials	0.335	0.101	0.722	0.107	0.491	0.138	0.801	0.115
Complex course contents	0.221	0.030	0.535	0.082	0.355	0.102	0.612	0.109
Inadequate support from lecturers	1.221	0.801	1.521	0.095	1.302	0.803	1.837	0.110
Limited time	0.752	0.315	1.305	0.112	1.991	0.776	2.183	0.146
No conducive environment (rf)								

*Source: Field Data (2019)*

## Discussions

The study aimed at developing and testing the effect of integrated conflict resolution lesson materials in a problem-based pedagogy on enhancing self-directed learning readiness among undergraduate nursing students enrolled in higher training institutions in Tanzania. The post-test findings revealed that the majority of nursing students who were exposed to conflict resolution lesson materials using facilitation in a problem-based pedagogy demonstrated the required levels of SDL readiness. Nursing students were able to demonstrate self-management over their learning activities by developing learning schedules including plans for searching additional academic resources and organizing peer groups for finding pedagogical strategies to address the presented problems.

Moreover, nursing students showed a desire for learning by asking and answering questions about some aspects that seem to be unclear to them. They were enthusiastic and excited about seeking more clarifications from facilitators and other relevant sources such as books to solve the assigned problems. What’s more, the study findings uncovered that nursing students demonstrated self-control over their learning activities by limiting other extracurricular activities and engaging in performing some simple tasks which were assigned to them as homework to be presented in the next session. Tasks such as storytelling that relate to conflicts and how they were solved and or finding intrinsic and extrinsic drivers of conflicts alongside possible strategies to address them. They were able to not only finish the assigned tasks within the allocated time but also making sure that they addressed them as per instructions and requirements provided by the facilitator.

These findings may imply that there was a strong positive link between the adoption of facilitation in a problem-based pedagogy and the overall SDL readiness and its associated domains among nursing students. The pedagogy demonstrated the academic potentials of influencing nursing students’ readiness to manage and control themselves in their learning activities that would positively catalyze their academic achievements. Interestingly, findings demonstrate that the use of innovative pedagogies in nursing education may hold a role in SDL readiness among nursing students such as empowering them to take responsibilities for their learning processes by identifying their learning objectives from a course, setting learning goals, identifying appropriate learning strategies and resources and evaluating their learning achievements. Moreover, other factors such as students’ accommodation status may also have a positive influence on SDL readiness as nursing students who were accommodated and living within their institutions’ premises exhibited an interest in learning, and readiness to manage and control themselves during their learning activities.

The findings of this study match with the work done by Haukedal and colleagues who found that there is a significant association between the type and nature of pedagogies used to facilitate learning among nursing students and the learning habits that in an actual sense reflects acceptable students’ levels of SDR readiness [32, 33]. Findings of these studies shade light that nursing students’ learning habits can enhance positively with the adoption of innovative nursing education pedagogies such as problem-based pedagogy. Similarities of findings between the two studies may be attributed to study designs and population. It is possible to note that the implementation of innovative teaching and learning styles in nursing education can have a greater emphasis placed on nursing students working independently during students’ learning activities.

The levels of SDL readiness under innovative pedagogies in nursing education have also been observed by previous studies, which revealed that an autonomous learning environment has the potentials of developing nursing students to be tuned with self-directed learning habits compared to the conventional education environment [26, 34]. In line with the findings of this study, the previous study has noted that with collaborative pedagogies rooting from the social constructivism learning theories, students can demonstrate primary responsibility for planning, implementing, and evaluating efforts in their learning activities [35]. Despite the difference in the study population, study design, study approaches, and location of the study, a match of the findings may probably be enhanced by the study of the same outcome variable in the medical field.

On the other hand, the finding of this study revealed that nursing students who were accommodated by their training institutions were more times likely to demonstrate self-directed learning readiness than their counterparts who were not. Tallying with this study educators and scholars have noted that students living in-campus can practice time management, study, and share knowledge with colleagues very easily and thus, promote group cohesion as well as discussions [36–38]. Findings may inform that off-campus learning environments are not conducive for academic works because of external distractors such as environmental noises from roadside/clubs, lack of consistent learning facilities and equipment, and classrooms being too far from where students live.

## Conclusions

The findings of this study imply that the use of facilitation in a problem-based pedagogy promises to enhance students’ SDL readiness in nursing education. The role of the tested pedagogy can emphasize nursing students to adopt changes in the learning environment and remain updated with the ever-changing and expanding evidence-based nursing practices. The pedagogy demonstrates the academic and professional potentials of changing the spectrum of nursing competencies among nursing students and nurse tutors thus, the quality and cost-effective care provided to patients and clients. The findings of this study provide policymakers, program and curriculum developers, and academics with important baseline data in their curricula renewal or development so that they stretch their expertise by considering and adopting the transformed pedagogies in the nursing curriculum.

Nevertheless, findings give light to educators on the need to change their ways of teaching and managing nursing students by devoting their time and professional competencies to tune themselves into the transformed innovative pedagogies such as facilitation in a problem-based pedagogy to enhance self-directed learning among nursing students in Tanzania. As it has been tested in this study, the pedagogy may empower nurse tutors, instructors, and clinical instructors in diagnosing students’ learning abilities and needs, and assist them to reach their academic and professional achievement towards addressing the societal contemporary health needs.

## Data Availability

Data are available on request at walter.millanzi@udom.ac.tz or wcleo87@gmail.com because further analysis of other variables are being processed.

## Abbreviations

AOR: Adjusted Odds Ratio
CI: Confidence interval
FPBP: Facilitation in a Problem Based Pedagogy
PhD: Doctor of Philosophy
SD: Standard Deviation
SDL: Self-directed Learning
SDLRS: Self-directed Learning Readiness Scale
SPSS: Statistical Product for Service Solutions (SPSS
UDOM: The University of Dodoma
WHO: World Health Organization (WHO)
